# Supplementary material for: Women’s experiences with the use of traditional medicine during childbirth in selected areas of Zambia
Source: PLOS Glob Public Health. 2025 Nov 25;5(11):e0005503. doi: 10.1371/journal.pgph.0005503 (PMC12646455; doi:10.1371/journal.pgph.0005503)
Supplement: Attachment — Submitted filename: Response to the Reviewers.docx [file pgph.0005503.s001.docx]

University of Zambia,

School of Public Health,

Lusaka.

Zambia.

7^th^ October 2025

Dear Reviewers,

I hope this email finds you well,

I am writing to express my sincere gratitude for the opportunity to review our manuscript reference number PGPH-D-25-01444 and titled “Women’s Experiences with the Use of Traditional Medicine during Childbirth in Selected Areas of Zambia”.

We appreciate the time and effort invested by the reviewers in evaluating our work.

We have carefully considered the reviewers' comments and suggestions, and have made significant revisions to improve the quality and clarity of our manuscript. Our responses to the reviewers' comments are outlined in the table below:

**RESPONSES AND COMMENTS FROM THE REVIEWER AND ACTION MATRIX**

| Reviewer Number | COMMENT | LINE | ACTION |
| --- | --- | --- | --- |
| Reviewer # 1 | Incorrect spelling for Discussion | 392 | Corrected the Spelling and now reads Discussion |
| Reviewer # 1 | Incorrect spelling for sitz baths |  | Corrected the Spelling and now reads sitz baths |
| Reviewer # 1 | The important finding on line that "friends were helpful in instances where the pregnancy was illegal and disapproved of by the family" is not highlighted in the results section | 402 and 403 | It has been added to the result section as guided |
| Reviewer # 2 | Summary of the background in the abstract |  | Summary of the background section of the abstract included as guided |
| Reviewer # 3 | what do you mean by “rolling”? | 33 | Rolling basis has been removed |
| Reviewer # 3 | Is “excessive bleeding” mentioned in the Results-section of the paper? (if so, everything is fine, and I apologize for the comment). | 37 | Excessive bleeding appears in the results section under negative experiences. |
| Reviewer # 3 | 2014 is a long time ago – is it possible to find new data? | 60 | Newer reference added as guided  the latest reference has a much lower rate 322,000 (WHO, 2021). |
| Reviewer # 3 | You talk about “Traditional medicine” and “Alternative therapies” – what is the difference? | 71-76 | Alternative therapy is more broad and is deleted to focus only on Traditional medicine |
| Reviewer # 3 | What do you mean by “abuse” here? | 77 | The phrase has been replaced “by have a lot of influence“ |
| Reviewer # 3 | In general, about the introduction: The references are relevant and good, but it is more like a series of quotes than a flowing text. I suggest that you work a bit more with this part. Think more like “you are telling a story to make me read your paper – not just giving me some quotes”. |  | Some references have been removed and the introduction flows as guided |
| Reviewer # 3 | what do you mean by “rolling”? | 137 | Rolling basis has been removed |
| Reviewer # 3 | “translated and transcribed” – below you state it the other way round. What is correct and where does it fit best in? | 163 | Deleted from the Data collection section. And is correctly stated under the data analysis section |
| Reviewer # 3 | The “in-depth interviews” are not mentioned under “Data collection” – did you do any? With whom? Describe under “Data collection” if you did. | 165 | In-depth interviews (IDIs) were used to collect data from elderly women who supplied traditional medicine and has been added to the data collection section as guided |
| Reviewer # 3 | “Herbs were either put or drunk in tea…” put where? What do you mean? “… or taken just before leaving” – but taken how? | 206 | The phrase has been rewritten  As “Herbs were either mixed with water for the maternal woman to drink or the herbs were added to porridge for the woman to eat. woman were usually given traditional medicine before leaving home to go and deliver at the hospital” |
| Reviewer # 3 | or inserting herbs” Inserting where? | 246 |  |
| Reviewer # 3 | What do you mean by “…her friend is late the one who knew the tree”? | 293 | Rewritten as Unfortunately, that friend of mum died and she was the one who knew the traditional medicine tree |
| Reviewer # 3 | UTH? | 309 | Written in full as university Teaching Hospital |
| Reviewer # 3 | Why suddenly “Group 1”? is that pregnant women or suppliers of treatment? | 314 | The participants have been clearly stated as  Women Who Used (WWU) traditional medicine  Women Who Supply (WWS) traditional medicine |
| Reviewer # 3 | What is “Ba WZ”? | 345 | It has been deleted |
| Reviewer # 3 | Ba WZ again? | 377-8 | It has been deleted |
| Reviewer # 3 | “…match..” – did you mean “…march…”? | 386 | The correct terminology to use march and this has been explained |
| Reviewer # 3 | In general, about the discussion: you should not discuss any results not given in the “Results” section! The discussion needs to be rewritten to mirror the results. Where in the results do you find anything about “correctly classifying, describing and identifying herbal products (ref lines 396-7)? | 396-7 | The Discussion has been rewritten and mirrors the results section |
| Reviewer # 3 | All the numbers given in lines 410-6 are not in the Results so can not be discussed here and numbers or percentages are not the point in a qualitative paper. You repeat yourself a lot in the beginning of the discussion talking about “family and friends” several times. | 410-6 | The numbers have been rewritten and the component on family has been summarized. |
| Reviewer # 3 | where is the abbreviation TP defined? | 413 | The abbreviation is defined as it means Traditional practitioners |
| Reviewer # 3 | squats and “Sizibaths” are not mentioned in the results, so should not be discussed here. | 434 | It has been added to the result section |
| Reviewer # 3 | It is a good idea to “educate women about potential risks” but what do we KNOW about potential risks? | 444 | The potential risks according to various studies have been added |
| Reviewer # 3 | You need to comment on HOW the other studies are similar to or different from yours – not just mention them. | 448-460 | This has been included |
| Reviewer # 3 | are the results you discuss here actually mentioned in the Results-section? | 461-6 | They have been added to the results section |
| Reviewer # 3 | are the results you discuss here actually mentioned in the Results-section? | 467-71 | They have been added to the results section |
| Reviewer # 3 | This is the really important part to discuss. | 481-6 | This has also been discussed in the discussion section |
| Reviewer # 3 | what do you want to study in a clinical trial? | 501 | Was deleted from the conclusion section |

We believe that these revisions have strengthened our manuscript and addressed the concerns raised by the reviewers. We are confident that our work will make a valuable contribution to Maternal Health and Midwifery practice.

Once again, many thanks for the feedback that was provided and thank you for considering our manuscript. We look forward to the opportunity to publish our work in PLOS Global Public Health.

Yours sincerely

Wanga Zulu

(On behalf of co-authors: Joseph Mumba Zulu, Charles Michelo, and Choolwe Jacobs)
